# 4-Chloro­anilinium 2-carb­oxy­acetate

**DOI:** 10.1107/S1600536812025469

**Published:** 2012-06-13

**Authors:** Min-Min Zhao

**Affiliations:** a College of Chemistry and Chemical Engineering, Southeast University, Nanjing 210096, People’s Republic of China

## Abstract

In the title molecular salt, C_6_H_7_ClN^+^·C_3_H_3_O_4_^−^, the components are linked by N—H⋯O and O—H⋯O hydrogen bonds, leading to a two-dimensional network parallel to the *bc* plane. Weak C—H⋯O inter­actions are also observed.

## Related literature

For the structures and properties of related compounds, see: Chen *et al.* (2001 ▶); Wang *et al.* (2002 ▶); Xue *et al.* (2002 ▶); Huang *et al.* (1999 ▶); Zhang *et al.* (2001 ▶); Ye *et al.* (2008 ▶).
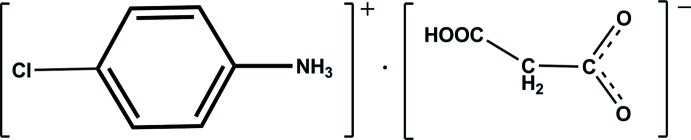


## Experimental

### 

#### Crystal data

C_6_H_7_ClN^+^·C_3_H_3_O_4_^−^
*M* *_r_* = 231.63
Monoclinic, 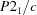

*a* = 12.8272 (19) Å
*b* = 9.2273 (10) Å
*c* = 8.4114 (13) Å
β = 93.809 (2)°
*V* = 993.4 (2) Å^3^
*Z* = 4
Mo *K*α radiation
μ = 0.38 mm^−1^
*T* = 153 K
0.10 × 0.05 × 0.05 mm

#### Data collection

Rigaku Mercury CCD diffractometer
Absorption correction: multi-scan (*CrystalClear*; Rigaku, 2005 ▶) *T*_min_ = 0.910, *T*_max_ = 1.000
6935 measured reflections
2259 independent reflections
1985 reflections with *I* > 2σ(*I*)
*R*_int_ = 0.025

#### Refinement

*R*[*F* ^2^ > 2σ(*F* ^2^)] = 0.034
*wR*(*F* ^2^) = 0.088
*S* = 1.06
2259 reflections
137 parameters
4 restraints
H-atom parameters constrained
Δρ_max_ = 0.32 e Å^−3^
Δρ_min_ = −0.27 e Å^−3^

### 

Data collection: *CrystalClear* (Rigaku, 2005 ▶); cell refinement: *CrystalClear*; data reduction: *CrystalClear*; program(s) used to solve structure: *SHELXS97* (Sheldrick, 2008 ▶); program(s) used to refine structure: *SHELXL97* (Sheldrick, 2008 ▶); molecular graphics: *SHELXTL* (Sheldrick, 2008 ▶); software used to prepare material for publication: *SHELXTL*.

## Supplementary Material

Crystal structure: contains datablock(s) I, global. DOI: 10.1107/S1600536812025469/bx2414sup1.cif

Structure factors: contains datablock(s) I. DOI: 10.1107/S1600536812025469/bx2414Isup2.hkl

Supplementary material file. DOI: 10.1107/S1600536812025469/bx2414Isup3.cml

Additional supplementary materials:  crystallographic information; 3D view; checkCIF report

## Figures and Tables

**Table 1:** Hydrogen-bond geometry (Å, °)

*D*—H⋯*A*	*D*—H	H⋯*A*	*D*⋯*A*	*D*—H⋯*A*
O4—H4⋯O1^i^	0.82	1.71	2.5314 (14)	176
N1—H1*B*⋯O2^ii^	0.89	1.87	2.7546 (16)	176
N1—H1*C*⋯O4^i^	0.89	2.25	2.8702 (15)	127
N1—H1*C*⋯O2^iii^	0.89	2.25	2.9313 (15)	133
N1—H1*A*⋯O3	0.89	1.91	2.7848 (15)	166
C3—H3*A*⋯O3	0.93	2.55	3.2599 (18)	134

Symmetry codes: (i) 
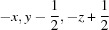
; (ii) 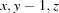; (iii) 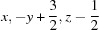.

**Fig. 1.**
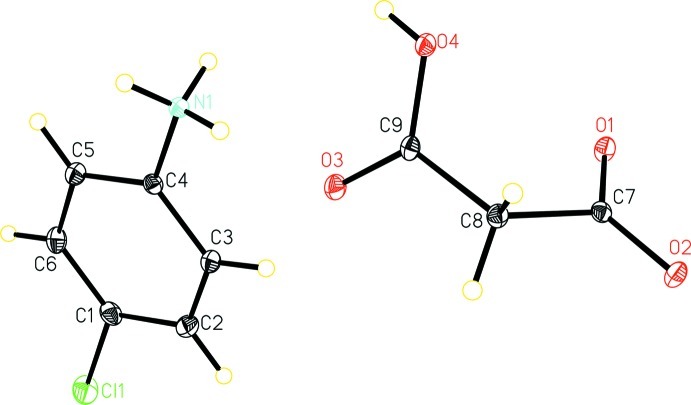
A view of the asymmetric unit with the atomic numbering scheme. The displacement ellipsoids were drawn at the 30% probability level.

## supplementary crystallographic information

### Comment

Simple organic salts containing strong intrermolecular H–bonds have attracted an attention as materials which display ferroelectric–paraelectric phase transitions (Chen *et al.*, 2001; Huang *et al.*, 1999; Zhang *et al.*, 2001). With the purpose of obtaining phase transition crystals of organic salts, various organic molecules have been studied and a series of new crystal materials have been elaborated (Wang *et al.*, 2002; Xue *et al.*, 2002; Ye *et al.* ,2008). Herewith, we present the synthesis and crystal structure of the title compound, 4-chloroanilinium 2–carboxyacetate.

In the title compound (Fig. 1), the bond lengths and angles have normal values. The asymmetric unit contains one 4-chloroanilinium cation and one 2–carboxyacetate anion. The protonated N atom is involved in strong intramolecular N—H···O hydrogen bonds with the N···O distances of N1—H1A···O3 = 2.7848 (15)Å; N1—H1B···O2 = 2.7546 (16)Å, N1—H1C···O2 = 2.9313 (15)Å and N1—H1C···O4 = 2.8702 (15)Å. The N—H···O and O—H···O H–bonding interactions connected the components into a 2D network parallel to the *bc*–plane. A weak non–classical intermolecular C3—H3A···O3 interaction is observed , Table1.

### Experimental

The malonic acid (10 mmol), 4-chloroaniline (10 mmol) and ethanol (50 mL) were added into a 100 mL flask. The mixture was stirred at 333 K for 2 h, and then the precipitate was filtrated out. Colourless crystals suitable for X–ray diffraction were obtained by slow evaporation of the solution.

### Refinement

All the H atoms attached to C atoms were placed into the idealized positions and treated as riding with C—H = 0.93Å (aromatic) and C—H = 0.97Å (methylene) with *U_iso_*(H) = 1.2*U_eq_*(C). The H atoms based on N and O were placed into the calculated positions with the H—N = 0.89Å and H—O = 0.82Å and refined with *U_iso_*(H) = 1.5*U_eq_*(N and O).

### Crystal data

**Table:**

C_6_H_7_ClN^+^·C_3_H_3_O_4_^−^	*F*(000) = 480
*M**_r_* = 231.63	*D*_x_ = 1.549 Mg m^−^^3^
Monoclinic, *P*2_1_/*c*	Mo *K*α radiation, λ = 0.71073 Å
Hall symbol: -P 2ybc	Cell parameters from 2259 reflections
*a* = 12.8272 (19) Å	θ = 2.7–27.5°
*b* = 9.2273 (10) Å	µ = 0.38 mm^−^^1^
*c* = 8.4114 (13) Å	*T* = 153 K
β = 93.809 (2)°	Block, colourless
*V* = 993.4 (2) Å^3^	0.10 × 0.05 × 0.05 mm
*Z* = 4	

### Data collection

**Table:**

Rigaku Mercury CCD diffractometer	2259 independent reflections
Radiation source: fine-focus sealed tube	1985 reflections with *I* > 2σ(*I*)
Graphite monochromator	*R*_int_ = 0.025
Detector resolution: 13.6612 pixels mm^-1^	θ_max_ = 27.5°, θ_min_ = 2.7°
CCD profile fitting scans	*h* = −16→16
Absorption correction: multi-scan (*CrystalClear*; Rigaku, 2005)	*k* = −9→11
*T*_min_ = 0.910, *T*_max_ = 1.000	*l* = −10→10
6935 measured reflections	

### Refinement

**Table:**

Refinement on *F*^2^	Primary atom site location: structure-invariant direct methods
Least-squares matrix: full	Secondary atom site location: difference Fourier map
*R*[*F*^2^ > 2σ(*F*^2^)] = 0.034	Hydrogen site location: inferred from neighbouring sites
*wR*(*F*^2^) = 0.088	H-atom parameters constrained
*S* = 1.06	*w* = 1/[σ^2^(*F*_o_^2^) + (0.0471*P*)^2^ + 0.2506*P*] where *P* = (*F*_o_^2^ + 2*F*_c_^2^)/3
2259 reflections	(Δ/σ)_max_ < 0.001
137 parameters	Δρ_max_ = 0.32 e Å^−^^3^
4 restraints	Δρ_min_ = −0.27 e Å^−^^3^

### Special details

**Table:**

Geometry. All esds (except the esd in the dihedral angle between two l.s. planes) are estimated using the full covariance matrix. The cell esds are taken into account individually in the estimation of esds in distances, angles and torsion angles; correlations between esds in cell parameters are only used when they are defined by crystal symmetry. An approximate (isotropic) treatment of cell esds is used for estimating esds involving l.s. planes.
Refinement. Refinement of F^2^ against ALL reflections. The weighted R-factor wR and goodness of fit S are based on F^2^, conventional R-factors R are based on F, with F set to zero for negative F^2^. The threshold expression of F^2^ > 2sigma(F^2^) is used only for calculating R-factors(gt) etc. and is not relevant to the choice of reflections for refinement. R-factors based on F^2^ are statistically about twice as large as those based on F, and R- factors based on ALL data will be even larger.

### Fractional atomic coordinates and isotropic or equivalent isotropic displacement parameters (Å^2^)

**Table:**

	*x*	*y*	*z*	*U*_iso_*/*U*_eq_	
Cl1	0.58798 (3)	0.24099 (5)	0.04636 (6)	0.03142 (14)	
O1	0.05355 (8)	0.92647 (10)	0.25990 (11)	0.0154 (2)	
O2	0.12165 (8)	1.07190 (10)	0.45291 (12)	0.0164 (2)	
O3	0.16242 (8)	0.58923 (10)	0.41276 (12)	0.0176 (2)	
O4	−0.00722 (7)	0.64921 (10)	0.40573 (11)	0.0150 (2)	
H4	−0.0212	0.5744	0.3562	0.022*	
N1	0.16273 (9)	0.31425 (13)	0.27635 (14)	0.0144 (3)	
H1A	0.1611	0.3942	0.3353	0.022*	
H1B	0.1469	0.2379	0.3348	0.022*	
H1C	0.1165	0.3220	0.1930	0.022*	
C9	0.09274 (11)	0.67518 (15)	0.43794 (15)	0.0125 (3)	
C7	0.09565 (10)	0.95030 (15)	0.39848 (16)	0.0119 (3)	
C4	0.26723 (10)	0.29563 (15)	0.22045 (15)	0.0137 (3)	
C1	0.46232 (12)	0.26233 (17)	0.11081 (19)	0.0203 (3)	
C8	0.11608 (11)	0.82132 (15)	0.51033 (16)	0.0139 (3)	
H8A	0.1889	0.8233	0.5495	0.017*	
H8B	0.0743	0.8332	0.6014	0.017*	
C6	0.39676 (12)	0.14367 (17)	0.11557 (19)	0.0217 (3)	
H6A	0.4186	0.0533	0.0817	0.026*	
C5	0.29804 (12)	0.16053 (16)	0.17131 (17)	0.0186 (3)	
H5A	0.2532	0.0816	0.1755	0.022*	
C2	0.43096 (12)	0.39847 (17)	0.15827 (18)	0.0206 (3)	
H2A	0.4755	0.4776	0.1531	0.025*	
C3	0.33224 (11)	0.41483 (16)	0.21357 (17)	0.0174 (3)	
H3A	0.3098	0.5054	0.2459	0.021*	

### Atomic displacement parameters (Å^2^)

**Table:**

	*U*^11^	*U*^22^	*U*^33^	*U*^12^	*U*^13^	*U*^23^
Cl1	0.0211 (2)	0.0287 (3)	0.0460 (3)	0.00583 (15)	0.01430 (18)	0.00397 (18)
O1	0.0212 (5)	0.0124 (5)	0.0121 (5)	0.0001 (4)	−0.0013 (4)	−0.0001 (4)
O2	0.0213 (5)	0.0111 (5)	0.0167 (5)	−0.0022 (4)	0.0002 (4)	−0.0015 (4)
O3	0.0179 (5)	0.0131 (5)	0.0219 (5)	0.0021 (4)	0.0030 (4)	−0.0023 (4)
O4	0.0165 (5)	0.0108 (5)	0.0171 (5)	0.0002 (4)	−0.0019 (4)	−0.0018 (4)
N1	0.0143 (5)	0.0126 (6)	0.0161 (6)	0.0000 (4)	−0.0005 (5)	−0.0010 (4)
C9	0.0177 (6)	0.0113 (7)	0.0086 (6)	0.0002 (5)	0.0012 (5)	0.0032 (5)
C7	0.0105 (6)	0.0126 (7)	0.0129 (6)	0.0013 (5)	0.0028 (5)	−0.0005 (5)
C4	0.0141 (6)	0.0146 (7)	0.0122 (6)	0.0012 (5)	−0.0005 (5)	0.0002 (5)
C1	0.0154 (7)	0.0235 (8)	0.0223 (7)	0.0046 (6)	0.0043 (6)	0.0032 (6)
C8	0.0164 (6)	0.0122 (7)	0.0126 (6)	0.0007 (5)	−0.0011 (5)	−0.0008 (5)
C6	0.0251 (8)	0.0154 (8)	0.0249 (8)	0.0049 (6)	0.0032 (6)	−0.0008 (6)
C5	0.0205 (7)	0.0142 (8)	0.0210 (7)	−0.0005 (5)	0.0000 (6)	−0.0006 (5)
C2	0.0187 (7)	0.0187 (8)	0.0245 (8)	−0.0026 (6)	0.0028 (6)	0.0023 (6)
C3	0.0187 (7)	0.0131 (7)	0.0203 (7)	0.0011 (5)	0.0003 (6)	−0.0016 (5)

### Geometric parameters (Å, º)

**Table:**

Cl1—C1	1.7455 (16)	C4—C5	1.379 (2)
O1—C7	1.2709 (16)	C4—C3	1.384 (2)
O2—C7	1.2488 (17)	C1—C6	1.383 (2)
O3—C9	1.2238 (17)	C1—C2	1.386 (2)
O4—C9	1.3149 (16)	C8—H8A	0.9700
O4—H4	0.8200	C8—H8B	0.9700
N1—C4	1.4598 (17)	C6—C5	1.388 (2)
N1—H1A	0.8900	C6—H6A	0.9300
N1—H1B	0.8900	C5—H5A	0.9300
N1—H1C	0.8900	C2—C3	1.386 (2)
C9—C8	1.5015 (19)	C2—H2A	0.9300
C7—C8	1.5289 (18)	C3—H3A	0.9300
			
C9—O4—H4	115.8	C9—C8—C7	115.35 (11)
C4—N1—H1A	109.5	C9—C8—H8A	108.4
C4—N1—H1B	109.5	C7—C8—H8A	108.4
H1A—N1—H1B	109.5	C9—C8—H8B	108.4
C4—N1—H1C	109.5	C7—C8—H8B	108.4
H1A—N1—H1C	109.5	H8A—C8—H8B	107.5
H1B—N1—H1C	109.5	C1—C6—C5	119.42 (14)
O3—C9—O4	124.03 (13)	C1—C6—H6A	120.3
O3—C9—C8	121.60 (12)	C5—C6—H6A	120.3
O4—C9—C8	114.37 (11)	C4—C5—C6	119.25 (14)
O2—C7—O1	125.35 (12)	C4—C5—H5A	120.4
O2—C7—C8	116.31 (11)	C6—C5—H5A	120.4
O1—C7—C8	118.34 (12)	C1—C2—C3	118.99 (14)
C5—C4—C3	121.36 (13)	C1—C2—H2A	120.5
C5—C4—N1	119.37 (12)	C3—C2—H2A	120.5
C3—C4—N1	119.25 (12)	C4—C3—C2	119.60 (13)
C6—C1—C2	121.38 (14)	C4—C3—H3A	120.2
C6—C1—Cl1	119.71 (12)	C2—C3—H3A	120.2
C2—C1—Cl1	118.91 (12)		

### Hydrogen-bond geometry (Å, º)

**Table:**

*D*—H···*A*	*D*—H	H···*A*	*D*···*A*	*D*—H···*A*
O4—H4···O1^i^	0.82	1.71	2.5314 (14)	176
N1—H1*B*···O2^ii^	0.89	1.87	2.7546 (16)	176
N1—H1*C*···O4^i^	0.89	2.25	2.8702 (15)	127
N1—H1*C*···O2^iii^	0.89	2.25	2.9313 (15)	133
N1—H1*A*···O3	0.89	1.91	2.7848 (15)	166
C3—H3*A*···O3	0.93	2.55	3.2599 (18)	134

Symmetry codes: (i) −*x*, *y*−1/2, −*z*+1/2; (ii) *x*, *y*−1, *z*; (iii) *x*, −*y*+3/2, *z*−1/2.
